# Mt Everest trek causes impaired cardiac high energy phosphate metabolism and diastolic impairment

**DOI:** 10.1186/1532-429X-11-S1-O6

**Published:** 2009-01-28

**Authors:** Cameorn J Holloway, Andrew Murray, Lowri E Cochlin, Yaso Emmanuel, Denny ZH Levett, Oliver J Rider, Damian J Tyler, Matthew Robson, Jane M Francis, Hugh Montgomery, Michael PW Grocott, Stefan Neubauer, Kieran Clarke

**Affiliations:** 1 University of Oxford, Oxford, UK; 2 UCL, London, London, UK

**Keywords:** Phosphate, Magnetic Resonance Imaging, Cardiac Function, Stroke Volume, Cellular Mechanism

## Background

Cardiac function in normal subjects is altered by exposure to hypobaric hypoxia, yet the cellular mechanisms leading to such changes are unknown. We have examined the impact of sustained exposure to environmental hypobaric hypoxia, on cardiac function and energetics.

## Methods and results

**Figure 1:**
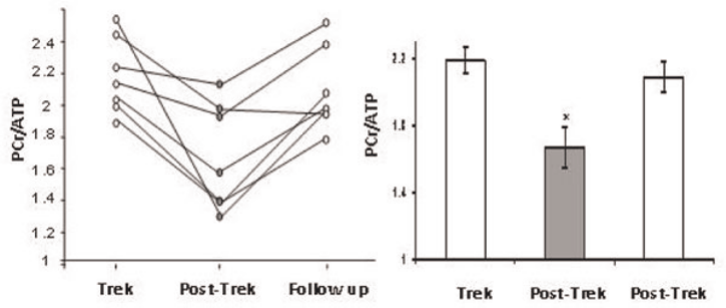
Figure 1

Healthy normal volunteers (n = 7) were studied immediately before, and within four days of return from 17 days exposure to environmental hypobaric hypoxia whilst trekking to Mount Everest Base Camp (17388 feet, 5300 m) and back. 31P magnetic resonance (MR) spectroscopy was used to measure cardiac phosphocreatine (PCr)/ATP, and MR imaging and echocardiography were used to assess cardiac function. All measurements were repeated six months after return from Everest. Immediately after their return from Everest, subjects showed a 24% decrease in cardiac PCr/ATP, from 2.19 ± 0.09 to 1.67 ± 0.13 (p < 0.01) (see Figure 1). Peak left ventricular filling rates had declined from 832 ± 64 ml/sec to 691 ± 56 ml/sec (p < 0.05) and transmitral E/A was reduced from 1.56 ± 0.11 to 1.16 ± 0.08 (p < 0.05). Left and right ventricular stroke volumes had fallen by 13%. No change on cardiac mass was observed. Six months later, all measures had returned to baseline values.

## Conclusion

Exposure to prolonged hypobaric hypoxia is associated with significant, but reversible, energetic and functional abnormalities in the human heart.

